# Muscle wasting associated with pathologic change is a risk factor for the exacerbation of joint swelling in collagen-induced arthritis in cynomolgus monkeys

**DOI:** 10.1186/1471-2474-14-205

**Published:** 2013-07-09

**Authors:** Naoto Horai, Takaharu Nagaoka, Itsuro Higuchi, Hayato Kasai, Takako Yoshioka, Yoshihisa Umekita, Koichiro Fukuzaki, Ryoichi Nagata, Atsuro Miyata, Kazuhiro Abeyama

**Affiliations:** 1 Department of Pharmacology, Kagoshima University Graduate School of Medical and Dental Sciences, 8-35-1 Sakuragaoka, Kagoshima 890-8544, Japan; 2 Shin Nippon Biomedical Laboratories, Ltd. Drug Safety Research Laboratories (SNBL DSR), 2438 Miyanoura, Kagoshima 891-1394, Japan; 3 Department of Neurology and Geriatrics, Kagoshima University Graduate School of Medical and Dental Sciences, 8-35-1 Sakuragaoka, Kagoshima 890-8544, Japan; 4 Department of Tumor Pathology, Kagoshima University Graduate School of Medical and Dental Sciences, 8-35-1 Sakuragaoka, Kagoshima 890-8544, Japan; 5 Division of Organ Pathology, Faculty of Medicine, Tottori University, 86 Nishi-machi, Yonago, Tottori 683-8503, Japan; 6 Department of Radiology, Morizono Hospital, 19-38 Oshoji-cho, Satsumasendai, Kagoshima 895-0076, Japan

**Keywords:** Collagen-induced arthritis, Rheumatoid arthritis, Cynomolgus monkey, Muscle wasting, Hypoxia, Steroid, Histopathology, Muscle

## Abstract

**Background:**

Not only joint destruction but also muscle wasting due to rheumatoid cachexia has been problem in terms of quality of life of patients with rheumatoid arthritis (RA). In the present study, we performed histopathological examination and assessed relationships between characteristic parameters relating to muscle and joint swelling in a collagen-induced arthritis (CIA) model using cynomolgus monkeys (CMs).

**Methods:**

Female CMs were used and CIA was induced by twice immunizations using bovine type II collagen with Freund’s complete adjuvant. Arthritis level was evaluated from the degree of swelling at the peripheral joints of the fore and hind limbs. Food consumption, body weight, and serum biochemical parameters were measured sequentially. Five or 6 animals per time point were sacrificed at 2, 3, 5 and 9 weeks after the first immunization to obtain quadriceps femoris specimens for histopathology. Pimonidazole hydrochloride was intravenously administered to determine tissue hypoxia in skeletal muscle.

**Results:**

Gradual joint swelling was observed and the maximum arthritis score was noted at Week 5. In histopathology, necrosis of muscle fiber in the quadriceps femoris was observed only at Week 2 and the most significant findings such as degeneration, atrophy, and regeneration of muscle fiber were mainly observed at Week 5. Food consumption was decreased up to Week 4 but recovered thereafter. Body weight decreased up to Week 5 and did not completely recover thereafter. A biphasic increase in serum cortisol was also observed at Weeks 2 and 5. Histopathology showed that muscle lesions were mainly composed of degeneration and atrophy of the muscle fibers, and ATPase staining revealed that the changes were more pronounced in type II muscle fiber than type I muscle fiber. In the pimonidazole experiment, mosaic pattern in skeletal muscle was demonstrated in the intact animal, but not the CIA animal. Increased arthritis score was accompanied by a decrease in serum creatinine, a marker that reflects muscle mass.

**Conclusions:**

Muscle wasting might exacerbate joint swelling in a collagen-induced arthritis model of cynomolgus monkeys.

## Background

We have already established a collagen-induced arthritis (CIA) model using female cynomolgus monkeys (CMs) and have assessed therapeutic and preventive effects of new drugs for rheumatoid arthritis (RA) in this model [1-3]. A treatment with biological agents targeting tumor necrosis factor-α (TNF-α) showed therapeutic effects in RA patients who were resistant to disease-modifying antirheumatic drugs (DMARDs) [4,5]. Employing primate models is considered to have many advantages for the prediction of drug effects with clinical application because of the similarities in drug metabolism between humans and CMs [6-8].

Rats and mice arthritis models induced by treatment with collagen (CIA) or adjuvant (AIA) are well known as animal models for chronic inflammation and RA [9-13]. However, it is difficult to assess toxicological and pharmacological aspects of biologics for humans with these rodent models, because rodent arthritis is not chronic, and swelling including soft tissue at peripheral part from wrist and ankle rather than joint structure itself is often assessed [14,15]. Additionally, humanized antibody drugs are easily rendered ineffective by rapidly produced neutralizing antibodies in rodents [16]. On the other hand, joint swelling in the CM CIA model is chronic, and the production of neutralizing antibodies for antibody drugs is negligible in CM in comparison with that in rodents [17,18].

Some epidemiological studies revealed a higher risk of cardiovascular disease [19,20] and muscle wasting [21,22] in RA patients compared with the general population. As well as the characteristic joint disorders, muscle dysfunction is a common and clinically intractable complication which also negatively affects prognosis and/or quality of life in RA [23,24]. The CIA model in CMs is also characterized by a body weight loss and cachexia associated with a loss of skeletal muscle.

RA is characterized by an inflammatory joint disease with chronic hypoxia and inflammatory cytokine production. Previous studies revealed hypoxia in the inflammatory tissues around the synovial membrane during RA development [25,26]. Although the linkage between hypoxia and inflammation is not clear, the possibility that tissue hypoxia and its resultant extracellular high mobility group box 1 (HMGB1) play an important role in arthritis development has been indicated [27].

Glucocorticoids regulate muscle metabolism and a number of steroids are used for patients with diverse diseases including RA [28,29]. However, it is known that cortisol, a representative intrinsic glucocorticoid in humans and monkeys, is induced by inflammation, and that muscle wasting due to the increases in the steroids is a critical clinical issue [30].

We hypothesized that body weight/muscle loss accompanied by inflammatory changes may have a role in the development of joint swelling as a representative pathosis in CIA. In the present study, we examined joints and skeletal muscle histopathologically to characterize the “arthritis-associated myopathy” and investigated the relation between muscle wasting and joint swelling, a major symptom of CIA.

## Methods

### Animals

Twenty-seven female cynomolgus monkeys (CMs) (*Macaca fascicularis*) with ages of 3 to 5 years were obtained from Guangdong Scientific Instruments & Materials Import/Export Corporation (Guangzhou, China), Wing freight agent Co., Ltd. (Beijing, China), China National Scientific Instruments & Materials Import/Export Corporation (Beijing, China), and Gaoyao Kangda Laboratory Animals Science & Technology Co., Ltd. (Guangdong, China). Twenty-two CMs were used for sequential histopathology and a part of the quadriceps femoris was collected for special staining (NADH-TR and ATPase staining) from 3 of the animals that were necropsied at Week 5 after the first immunization. Additionally, arthritis was induced in 1 animal, which was then allocated for a pimonidazole dosing study. One intact animal was used for the pimonidazole dosing study as a control animal. The 22 CMs to be used for histopathology were weighed using an electronic balance (HP-40 K, A & D Co., Ltd.) once a week throughout the experiment. Approximately 108 g of solid food (Teklad Global Certified 25% Protein Primate Diet, Harlan Sprague Dawley Inc.) was provided to each animal daily. Food consumption was calculated daily from the amounts of food supplied and remaining, and averaged daily food consumption was calculated for each week. Water was available *ad libitum* from an automatic supply (Edstrom Industries, Inc.).

### Animal welfare

All procedures for animals were approved by the Intuitional Animal Care and Use Committee of SNBL and were performed in accordance with standards published by the National Research Council (Guide for the Care and Use of Laboratory Animals, NIH OACU) of the National Institutes of Health Policy on Human Care and Use of Laboratory Animals. Additionally, the animals used in this model received special treatments to moderate emaciation. In accordance with these standards, every effort was made to ensure that the subjects were free of pain and discomfort.

### Arthritis induction

Bovine type II collagen (CII) (4 mg/mL, Collagen Research Center, Tokyo, Japan) was used. The CII solution and Freund's complete adjuvant (FCA) (Becton Dickinson, Grayson, GA, USA) were mixed in equal proportions using a syringe. Each CM was anaesthetized by intramuscular injection of 10 mg/kg ketamine and intracutaneously injected 2 mL of the emulsion on the back. The second immunization with CII and FCA was conducted 3 weeks after the first immunization, in the same manner [1,2].

### Observation of swelling at the joints

Arthritis level was evaluated by monitoring the degree of swelling at the metacarpophalangeal, proximal interphalangeal, and distal interphalangeal joints, and the wrist, ankle, elbow, and knee (total 64 joints) at Weeks 2, 3, 4, 5, 7 and 9 after the first immunization. Each joint was assessed in accordance with the evaluation criteria shown as follows: Score 0, No abnormality; Score 1, Swelling not visible but can be determined by touch; Score 2, Swelling slightly visible and can be confirmed by touch; Score 3, Swelling clearly visible; Score 4, Rigidity of the joints. The arthritis score for each animal was designated as the total score of individual joints.

### Blood chemistry

Blood was drawn from the femoral vein at Weeks 2, 3, 4, 5, 7 and 9 after the first immunization, and serum was obtained by centrifugation (room temperature, 1710 *g* for 15 minutes). Creatine phosphokinase (CPK), creatinine and C-reactive protein (CRP) were determined by an automatic analyzer (JCA-BM8, JEOL Co., Ltd., Tokyo, Japan).

### Measurement of serum cytokines and cortisol

Serum interleukin-6 (IL-6), IL-2, IL-4, IL-5, TNF, and IFN-γ at Weeks 2, 3, 4, 5, 7 and 9 after the first immunization were determined with nonhuman primate Th1/Th2 Cytokine CBA kit (BD Biosciences, San Diego, CA, USA). Cortisol concentrations in serum obtained in the morning on each day were measured by radioimmunoassay with DPC-Cortisol kit (Siemens Healthcare Diagnostics, Los Angeles, CA, USA).

### Histopathology

At Weeks 2, 3, 5 or 9 after the first immunization, 5 or 6 animals/time point were euthanized by exsanguination under anesthesia by an intravenous injection of a solution of sodium pentobarbital (Tokyo Chemical Industry Co., Ltd., 64.8 mg/mL, 0.4 mL/kg) and necropsied. The quadriceps femoris muscle from the center of lateral vastus on the left side was collected from each animal, and a half of the collected tissue was fixed in 10 v/v% neutral buffered formalin and the remainder was quickly frozen in liquid nitrogen. The formalin-fixed tissue samples were embedded in paraffin, sectioned and stained with hematoxylin-eosin (HE). For electron microscopy, small pieces of the formalin-fixed muscle from 1 CIA animal with marked histopathological lesions were re-fixed with 3% glutaraldehyde and followed by a double fixation with 1% osmium tetroxide. Ultra-thin sections were prepared and double-stained with uranyl acetate and lead citrate, and examined with a transmission electron microscope (JEM-1200EX, JEOL Co., Ltd.). NADH-TR and ATPase staining was performed with cryosections of muscle collected at Week 5.

### Detection of hypoxia in the skeletal muscle with pimonidazole

Hypoxic changes in the left quadriceps femoris were visualized using a hydroxyprobe-1 kit (Chemicon International, Temecula, CA, USA) as follows. Pimonidazole hydrochloride, a novel hypoxic marker agent, was dissolved in sterile physiological saline and 2 monkeys (one was a CIA model animal and the other an intact control animal) were administered intravenously with the pimonidazole solution at a dose of 0.5 g/m^2^ at Week 5. At 18 hours after dosing, the animals were euthanized by exsanguination under anesthesia by an intravenous injection of the solution of sodium pentobarbital (0.4 mL/kg). A piece of the quadriceps femoris muscle was collected from each animal, fixed in 10 v/v% neutral buffered formalin, and embedded in paraffin. A hydroxyprobe-1 monoclonal antibody was used to stain pimonidazole adducts in the thin-sectioned tissues in accordance with the recommendations of the manufacturer.

### Statistical analysis

**Figure 1:**
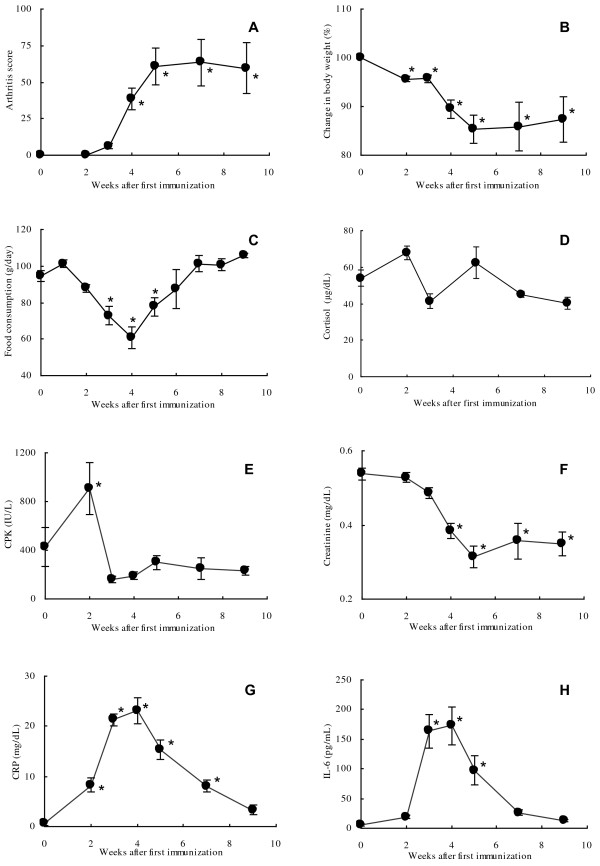
**Sequential changes of the parameters in the process of arthritis induction.** Changes in arthritis score **(A)**, body weight **(B)**, food consumption **(C)**, serum cortisol concentration **(D)**, creatine phosphokinase **(E)**, creatinine **(F)**, C-reactive protein **(G)**, and interleukin-6 **(H)**. Values are expressed as the mean ± SEM. Differences between post- and pre-dosing values were statistically examined using a repeated measures ANOVA model. Statistical differences were evaluated at 5%. N = 22 at pre-immunization and Weeks 1 and 2, N = 17 at Week 3, N = 12 at Weeks 4 and 5, and N = 6 at Weeks 6 to 9. Change in body weight (%) was calculated as the percentage of weight relative to the weight at pre-immunization.

**Figure 2:**
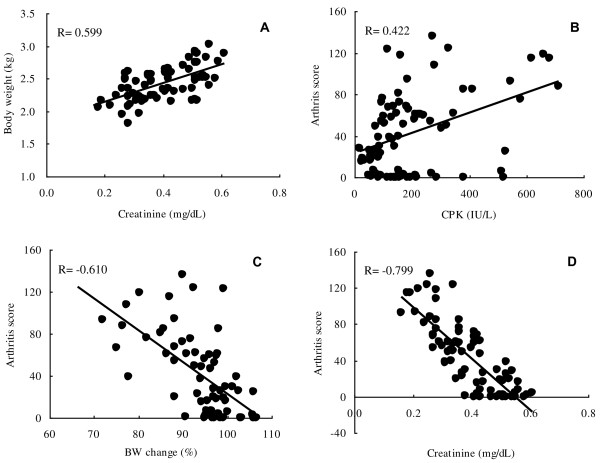
**Correlation analysis.** Relationships between serum creatinine level and body weight **(A)**, creatine phosphokinase and arthritis score **(B)**, change in body weight and arthritis score **(C)**, and serum creatinine level and arthritis score **(D)**. Pearson’s correlation coefficients between the parameters (arthritis score, serum creatinine, serum CPK, and body weight) were calculated. The concentration of serum creatinine, a marker to reflect muscle mass, was positively correlated with body weight **(A)**. CPK levels were not well related with arthritis score **(B)**, but arthritis score showed a weak correlation with body weight change **(C)**. Arthritis score showed a strong correlation with creatinine levels **(D)**.

Values are presented as the mean ± SE. Differences between post- and pre-dosing values as presented in Figure 1 were statistically examined using a repeated measures ANOVA model. Statistical differences were evaluated at 5%. The number of animals at each time point were as follows; N = 22 at pre-immunization and Weeks 1 and 2, N = 17 at Week 3, N = 12 at Weeks 4 and 5, and N = 6 at Weeks 6 to 9. Pearson’s correlation coefficients were calculated for parameters (arthritis score, serum creatinine, serum CPK, and body weight) as shown in Figure 2.

## Results

### Arthritis induction and its characters

Joint swelling was observed mainly at the fingers of right and left hands and feet after CII immunization. There were individual differences in the time of onset and severity of the swelling. The mean arthritis score increased from Week 3 and was elevated sequentially with a peak value of 68.9 at Week 7, and the score was maintained around the peak value until Week 9 (Figure 1A). X-ray findings such as joint space narrowing, bone atrophy, and architectural joint destruction were aggravated time-dependently until Week 9 (data not shown).

### Body weight and food consumption

Accompanying the occurrence of arthritis, body weights decreased markedly at Week 4 and remained lower than the value at pre-immunization until Week 9 (Figure 1B). Food consumption decreased time-dependently from the early phase of the induction period, and the most significant decrease in body weight was noted at Week 4, but food consumption recovered from the decrease to within the range of pre-immunization period by Week 7 (Figure 1C). The timing of recovery of food consumption was slightly sooner than that of body weight, and the time of transition was not consistent between these two parameters in the later phase of the induction period. These results support the previous observations that demonstrated that cachexia is not the result of a decrease in caloric intake [21].

### Blood chemistry and serum cytokine levels

CPK was increased markedly at Week 2; however, the increase was transient and was followed by a decrease to the pre-immunization level (Figure 1E). Creatinine decreased gradually from the early phase of induction period and reached nadir at Week 5, and no marked increase was noted until Week 9 (Figure 1F). Creatinine is known to be a marker which reflects muscle mass and to correlate with body weight. Both CRP and IL-6 increased up to Week 4, and then decreased time-dependently (Figures 1G, H). Serum TNF-α level was below the lower limit of quantification (20 pg/mL) in all animals at all time points, and no changes that were considered to be related to onset of arthritis were noted in the other cytokines (data not shown).

### Histopathological features

**Figure 3:**
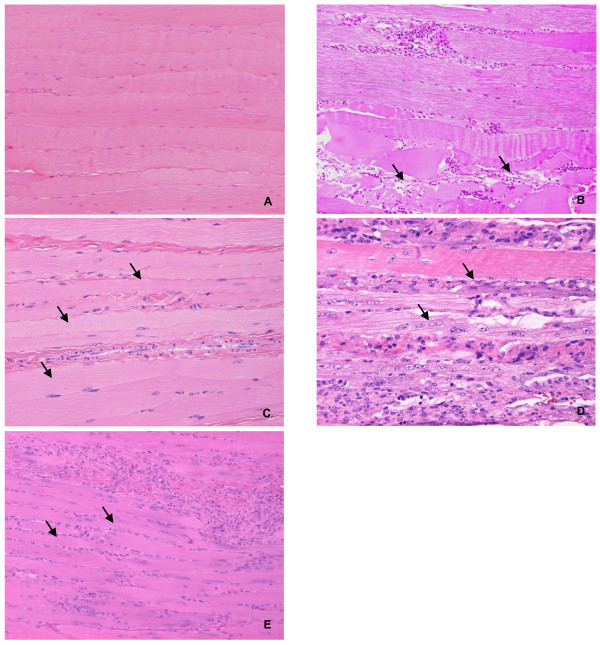
**Sequential changes of histopathologic feature of quadriceps femoris in HE staining. A**: naive animal, **B**: necrosis and degeneration (↑) of muscle fiber and inflammatory cell infiltration at Week 2, **C**: degeneration and atrophy (↑) of muscle fiber at Week 3, which is clearer than that at Week2, **D**: regeneration (↑) of muscle fiber at Week 5, **E**: regeneration and atrophy (↑) at Week 9 similar to, but a lesser degree to that at Week 5 **(E)**.

**Figure 4:**
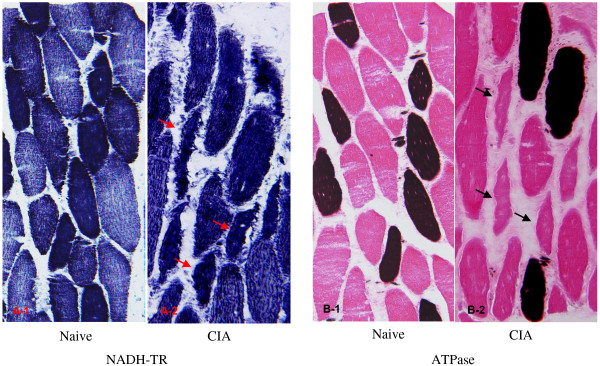
**Histopathologic feature of quadriceps femoris in NADH-TR and ATPase staining.** NADH-TR and ATPase staining in a naive animal **(A-1** and **B-1)** and a CIA animal **(A-2** and **B-2)**. In NADH-TR stain, a clear difference in stainability between muscle fibers reflecting the classification into type I and type II muscle fibers was observed in the naive animal; however, a difference in stain ability was not shown and irregularity in the size of muscle fibers was observed in the CIA animal. ATPase staining in CIA animal showed that the atrophic changes in the type II muscle fibers were more significant than those in the type I muscle fibers.

**Figure 5:**
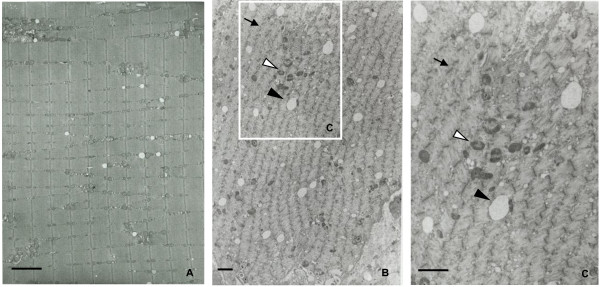
**Electron microscopy in quadriceps femoris in naive and CIA animals.** Irregularity of Z-bands (↑) and increases in the number of mitochondria (△) and small vesicles (▲) were observed in the CIA animal **(B)** in comparison with a naive animal **(A)**. **C** is high magnification of the region enclosed in the square in **B**. (bar 2 μm).

The incidences of histopathological changes in the muscle fiber of the quadriceps femoris at each time point are summarized in Table 1. Degeneration, necrosis, or regeneration of muscle fiber was diagnosed, when a few mass or affected muscle bundles was observed in several scattered areas. Atrophy was diagnosed when width of the muscle bundle was smaller than half width of normal bundle. Necrosis and degeneration of the muscle fiber were seen in 2 and 1 of 5 animals at Week 2, respectively, but the incidences of degeneration and atrophy increased time-dependently until Week 5. Regeneration was seen at Week 5 and thereafter (Figures 3A to E). In NADH-TR staining, there was a difference in stain ability between the muscle fiber types (intensely stained: type I muscle fiber, slightly stained: type II muscle fiber) in a naive animal (Figure 4A-1); however, the difference in stain ability was lost and irregularity in the size of muscle fibers was observed in the CIA animal (Figure 4A-2). ATPase staining showed that atrophy of type II muscle fiber was predominant when compared to that of the lesion in type I muscle fiber (Figure 4B-1: naive animal, and Figure 4B-2: CIA animal). In electron microscopic observation, the number of mitochondria and vesicle increased with irregular alignment as the most characteristic change (Figures 5B, C) compared with a naive animal (Figure 5A).

**Table 1:** Frequency and percentage of animals with histopathological findings in muscle fiber

	**Week 2**		**Week 3**		**Week 5**		**Week 9**	
	**(N = 5)**		**(N = 5)**		**(N = 6)**		**(N = 6)**	
	**#**	**%**	**#**	**%**	**#**	**%**	**#**	**%**
Necrosis	2	40	1	20	0	0	0	0
Degeneration	1	20	3	60	6	100	5	83
Atrophy	0	0	3	60	4	67	4	67
Regeneration	0	0	0	0	3	50	1	17

#: Number of the animals with the histopathological finding.

%: Percentage of the animals with the histopathological finding

As for degeneration, necrosis, or regeneration of muscle fiber, when a few mass or affected muscle bundles were observed in several scattered areas, the finding was judged positive. Atrophy was diagnosed when width of the muscle bundle was smaller than half width of normal bundle. The number of animals with the above lesions was summarized in Table 1.

### Detection of hypoxic change in femoral muscle

**Figure 6:**
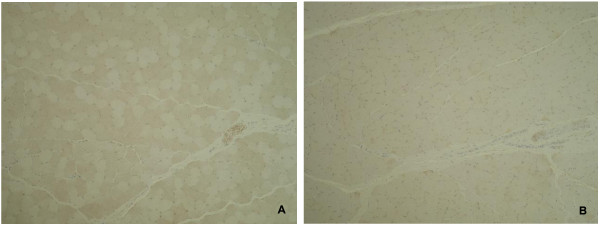
**Immunostaining for pimonidazole dosing experiment in quadriceps femoris in naive and CIA animals.** Mosaic pattern attributable to distribution of type I and II muscle fibers was evident in a naive animal **(A)**; conversely, no similar mosaic pattern was observed in a CIA animal **(B)**.

The pimonidazole staining of the quadriceps femoris from 1 immunized and 1 non-immunized CM demonstrated a mosaic pattern of skeletal muscle in the naive animal (Figure 6A), and this was considered to reflect the normal distribution of type I (aerobic: unstained) and II (anaerobic: stained) muscle fibers. On the other hand, no mosaic pattern was observed in the quadriceps femoris of the CIA animal (Figure 6B), indicating that the normal distribution of the fibers had been changed in the muscle.

### Serum cortisol level

Serum cortisol levels were elevated at Week 2 and dropped below the baseline at Week 3, and then were elevated again at Week 5. The biphasic increase was considered attributable to twice immunizations (Weeks 0 and 3) with type II collagen. Thereafter, levels gradually decreased until Week 9 (Figure 1D).

### Relationships between arthritis score and muscle-related blood parameters

Relationships between severity of swelling at the joints and blood parameters related to muscle (i.e. creatinine and CPK) were investigated. Serum creatinine is considered to depend on muscle mass, and its concentrations were positively correlated with body weight (R = 0.599, Figure 2A). Although CPK levels were not well related with arthritis score (R = 0.422, Figure 2B), arthritis score showed a weak correlation with body weight change [body weight change was calculated with the following formula: body weight change (%) = body weight at each week (kg)/body weight before immunization (kg) × 100] (R = −0.610, Figure 2C) and a stronger correlation with creatinine levels (R = −0.799, Figure 2D).

## Discussion

In patients with RA, not only pain and dysfunction at joints but also cachexia are observed frequently [31-33], and rheumatoid cachexia is still remains one of major issues. Multiple factors such as disuse atrophy due to joint pain and proinflammatory cytokines such as TNF-α, IL-1β, and IL-6 are considered attributable to rheumatoid cachexia; however, there have been few reports regarding histopathology of muscle in RA patients and the cause of rheumatoid cachexia is not completely understood yet. In the present study, we sequentially assessed histopathological muscle lesions in a CIA model using CMs. In addition, we investigated the relationships between joint swelling, a major symptom of arthritis, body weight, and blood chemistry parameters relating to muscle.

In histopathology of HE stained specimens of the quadriceps femoris, necrosis of muscle fibers was observed only in 2 and 1 of 5 animals at Weeks 2 and 3, respectively, and degeneration and atrophy were observed in almost all animals from Week 5. Additionally, similar findings were observed in biceps brachii muscle, longissimus muscle, and gastrocnemius (data not shown), indicating that atrophic change in muscle was induced systemically as shown by the decrease in body weight. Food consumption almost recovered to the range of the values at pre-immunization by Week 9, but body weight did not completely recover by that time, showing that the decrease in body weight including these histopathological lesions were not considered to be attributable only to malnutrition. In NADH-TR staining, a clear difference in stain ability between muscle fibers reflecting the classification into type I and type II muscle fibers was observed in a naive animal; however, the difference in stain ability was not shown in the CIA animals. Additionally, ATPase staining showed a significant atrophic lesion in the type II muscle fibers but not in the type I muscle fibers. These changes would be related to the disappearance of normal mosaic pattern (that would reflect the regular distribution of the type I and type II muscle fibers) in CIA model which was confirmed in the pimonidazole dosing experiment, and indicated that the normal distribution of partial pressure oxygen had been lost in CIA model. In addition, electron microscopic observation revealed an increase in the number of mitochondria, and this was considered to be a compensatory reaction against hypoxia. The histopathological lesions in the muscle observed in the present study were similar to those of the patients with RA [34]. The study of hypoxia in RA patients showed that hypoxia and HMGB-1 were associated with development of arthritis [27]. On the other hand, physical activity and aerobic exercise ameliorate not only the motor function but also swelling and stiffness of the joints in clinical practice. These reports showed that hypoxia was associated with swelling and malfunction of joints, at least in part. Moreover, the results in the present study indicated that muscle hypoxia was possibly related to the muscle wasting.

It is known that muscle atrophy can be mediated by multiple factors, including glucocorticoids [28-35], and proinflammatory cytokines such as IL-1β, TNF-α, and IL-6 [35]. Previous studies showed that the expression and activity of the transcription factor C/EBPβ and δ were increased in the skeletal muscle during sepsis in rats [36]. In the same experiments, treatment with the glucocorticoid receptor antagonist prevented the sepsis-induced activation of C/EBPβ and δ, suggesting that the transcription factors are, at least in part, regulated by glucocorticoids. The expression of these transcription factors, especially that of C/EBPβ, was confirmed by western-blot analysis in skeletal muscle obtained from the present study, and it was most significant at Week 5 (data not shown), and a marked increase in serum cortisol concentration was noted at the same time point. These results indicated that cortisol is involved in an increase in expression of C/EBPβ and might be related to the muscle wasting in this CIA model.

Although serum TNF-α was not detected at any time point, significant increases in glucocorticoid and proinflammatory cytokines such as IL-6 were noted in this model, and these findings were consistent with results from clinical practice [37]. Further research is needed to elucidate the direct relationship between hypoxia, glucocorticoid, and proinflammatory cytokines in skeletal muscle since it was not made clear in the present study.

The prevalence of cardiovascular disease (CVD) in patients with RA is higher [38], and the risk of mortality due to CVD is 50% higher [39] than in the general population. As chronic systemic inflammation has been reported to be related to CVD [40,41], the heart was also examined histopathologically in the present study, but no abnormality was observed in this organ in any animal in the present study (data not shown). These results showed that the degree of inflammation noted in this CIA model was not associated with CVD. Generally, the creatinine level in preserved urine is measured for an indirect assessment of muscle quantity in clinical practice [31]. In CMs, the volume of urine depends only on spontaneous urination and is unstable; therefore, serum creatinine was measured instead of urine volume in the present study. The results showed that a positive correlation (R = 0.599) was noted between body weight and serum creatinine level. The percentage of body fat was about 5% in CMs of medium-size, as quantified with the DXA method (data not shown), which is not higher than that in humans. Serum creatinine could be as accurate an indicator of muscle quantity in CMs as it is in humans.

Arthritis score did not show a clear correlation with serum CPK (R = 0.422); however, the score showed a negative correlation with the change in body weight based on the value at pre-immunization (R = −0.610) and a stronger negative correlation with serum creatinine, a marker which reflects muscle mass (R = −0.799). Thus, arthritis score was increased accompanied by a decrease in serum creatinine, a marker to reflect muscle mass, and the results were considered plausible and capable of extrapolation to humans since aging is thought to be one of the risk factors for RA and muscle mass generally decreases with aging. Also in the study using mice CIA model, a similar correlation was noted between muscle weight and arthritis score [42].

## Conclusions

From these results, we concluded that muscle wasting exacerbates joint swelling, and that therapeutic target would not only be the arthritic joints but also the skeletal muscle. The CIA model using CMs has similar characteristics to patients with RA in respect of the changes at the joints and in muscle, suggesting that the CIA monkey model in the present study can be useful for the development of new drugs for human arthritis therapy.

## Abbreviations

CIA: Collagen-induced arthritis; CM: Cynomolgus monkey; RA: Rheumatoid arthritis; TNF-α: Tumor necrosis factor-α; DMARDs: Disease-modifying antirheumatic drugs; AIA: Adjuvant-induced arthritis; CII: Bovine type II collagen; FCA: Freund's complete adjuvant; CPK: Creatine phosphokinase; CRP: C-reactive protein; IL-6: Interleukin-6; HE: Hematoxylin-eosin; CVD: Cardiovascular disease.

## Competing interests

The authors declare that they have no competing interests.

## Authors’ contributions

Abeyama contributed to the study design. Horai, Nagaoka, Higuchi, Kasai, Yoshioka, and Umekita contributed to the acquisition of data. Horai, Nagaoka, Higuchi, Fukuzaki, Nagata, Miyata, and Abeyama contributed to the analysis and the interpretation of data. Horai, Miyata, and Abeyama contributed to the manuscript preparation. All authors read and approved the final manuscript.

## Pre-publication history

The pre-publication history for this paper can be accessed here:

http://www.biomedcentral.com/1471-2474/14/205/prepub
